# Patient-Centred Care for Patients With Diabetes and HIV at a Public Tertiary Hospital in South Africa: An Ethnographic Study

**DOI:** 10.34172/ijhpm.2020.65

**Published:** 2020-05-06

**Authors:** Edna N. Bosire, Emily Mendenhall, Shane A. Norris, Jane Goudge

**Affiliations:** ^1^South African Medical Research Council Developmental Pathways for Health Research Unit (DPHRU), School of Clinical Medicine, Faculty of Health Sciences, University of the Witwatersrand, Johannesburg, South Africa.; ^2^Science, Technology, and International Affairs Program, Walsh School of Foreign Service, Georgetown University, Washington, DC, USA.; ^3^School of Human Development and Health, University of Southampton, Southampton, UK.; ^4^Centre for Health Policy, School of Public Health, Faculty of Health Sciences, University of the Witwatersrand, Johannesburg, South Africa.

**Keywords:** Diabetes, HIV/AIDS, Patient-Centred Care, South Africa

## Abstract

**Background:** Healthcare systems across the globe are adopting patient-centred care (PCC) approach to empower patients in taking charge of their illnesses and improve the quality of care. Although models of patient‐centredness vary, respecting the needs and preferences of individuals receiving care is important. South Africa has implemented an integrated chronic disease management (ICDM) which has PCC component. The ICDM aims to empower chronic care patients to play an active role in disease management process, whilst simultaneously intervening at a community/ population and health service level. However, chronic care is still fragmented due to systemic challenges that have hindered the practice of PCC. In this article, we explore provider perspectives on PCC for patients with comorbid type 2 diabetes and HIV at a public tertiary hospital in urban South Africa.

**Methods:** This study utilizes ethnographic methods, encompassing clinical observations, and qualitative interviews with healthcare providers (n=30). Interview recordings were transcribed verbatim and data were analyzed inductively using a grounded theory approach.

**Results:** Providers reported various ways in which they conceptualized and practiced PCC. However, structural challenges such as staff shortages, lack of guidelines for comorbid care, and fragmented care, and patient barriers such as poverty, language, and missed appointments, impeded the possibility of practicing PCC.

**Conclusion:** Health systems could be strengthened by: *(i)* ensuring appropriate multidisciplinary guidelines for managing comorbidities exist, are known, and available, *(ii)* strengthening primary healthcare (PHC) clinics by ensuring access to necessary resources that will facilitate successful integration and management of comorbid diabetes and HIV, *(iii)* training medical practitioners on PCC and structural competence, so as to better understand patients in their sociocultural contexts, and *(iv)* understanding patient challenges to effective care to improve attendance and adherence.

## Background

The number of people that require chronic disease care is projected to increase in sub-Saharan Africa as a result of expanding HIV treatment coverage, rising life expectancies,^1^ and a rapid increase of non-communicable diseases (NCDs).^2^ This trend is expected to continue challenging today’s health systems, particularly as the shift in the burden of disease increases the need for people with multi-morbidities to have continuing contact with multiple practitioners in the health system.^3^ HIV and NCDs require ongoing attendance at appointments, adherence to tests and medications, healthy living and self-management. This is exemplified by diseases, such as type 2 diabetes (hereafter, diabetes) that require constant glucose monitoring, healthy eating, foot care, and exercise that involve active self-management away from the formal health system. As a result, healthcare systems are adopting integrated and patient-centred practices that aims to deliver quality care and value for money, while acknowledging the role that patients play in chronic care.

In South Africa, convergence of chronic diseases such as HIV and diabetes are now becoming common and are overburdening the healthcare system.^4^ This reflects the high prevalence of HIV in the country, estimated at 13% of the total population^5^ and a rapid increase of NCDs including diabetes.^6^ One study in a South African peri-urban settlement found that one in 4 patients seeking primary care had multi-morbidities and that 45% of adults sought prescriptions for HIV, tuberculosis, diabetes, and/or hypertension.^4^ Recently, a study conducted in rural settings in South Africa has reported a rapid increase of patients with multi-morbidities, exemplified by a clustering of cardiometabolic conditions and HIV.^7^ Simultaneously, most South Africans afflicted by multiple morbidities rely exclusively upon the public health system for detection, treatment, and care.^8^ Yet, the state of public hospitals is overburdened: healthcare personnel are inadequately trained, there is erratic drug supply and frequent stock outs, and resource allocation of finances and essential equipment is inadequate.^9^ The staff shortages especially hinder providers’ abilities to develop relationships with patients and impede long term care.^10^ This may in part be exacerbated when differences between patients and providers, such as race and class, make it more difficult to understand one another.^11,12^ Thus, there is need to improve patients’ access to care, reduced cost of care and improve patient-provider relationships by adopting integrated and patient-centred care (PCC) models.

There is no unifying definition or common conceptual understanding of integrated care, but integration involves a set of methods and models for the funding, administrative, organization, service delivery, and clinical levels designed to create connectivity, alignment, and collaboration between the cure and care sectors.^13^ The World Health Organization (WHO) defines integrated healthcare as “the organization, management and coordination of health services so that people get the care they need, when they need it, in ways that are user-friendly, achieve the desired results and provide value for money.”^14^ Following evidence that integrated chronic disease care improves patient health outcomes,^15^ the Joint United Nations Programme on HIV/AIDS (UNAIDS) recommended an integrated approach for chronic disease management. This approach leverages the innovations of the HIV programme to support or scale up services for NCDs^16^ using the WHO’s building blocks described in the Innovative Care for Chronic Conditions (ICCC) framework.^17^ The ICCC emphasizes the importance of offering quality healthcare services, the integration of chronic conditions services, and adaptability to changes in burden of disease.^17^

In South Africa, the ICCC was adapted through formulation of the integrated chronic disease management (ICDM) framework between 2011 and 2013.^18^ This framework is based on a Public Health approach to empower the individual to take responsibility for their own health, whilst simultaneously intervening at community/population and health service levels. The ICDM argues that optimal clinical outcomes for people living with single or multi-morbid conditions can be achieved through primary healthcare (PHC) re-organization involving improved clinical management support, clinical practice guidelines for integrated care, and the use of community healthcare workers to assist patients with self-management.^18^ In addition, unlike the HIV care models that are vertical in nature, the ICDM adopts a diagonal approach to health system strengthening, ie, technical interventions that improve the quality of care for chronic patients coupled with the strengthening of the support systems and structures to enhance the health system.^18^ Thus, ICDM is centred around service users, and it forms the core foundation in providing PCC.

There is no universally accepted definition of PCC,^19^ but there is general agreement that it broadens the conventional medical approach to include the patient as an active participant in his or her care, and to promote the physician-patient partnership.^20^ PCC is a philosophy built around the needs of the individual and contingent upon knowing the person through an interpersonal relationship.^21^ In this way, patients must also understand their role as partners in care and be willing to collaborate with providers as well as share their self-care experiences and concerns. This requires patients to acquire adequate knowledge, motivation, skills, and confidence^22,23^ to participate in their own care and manage their conditions, a concept known as ‘patient activation.’^24^

A shift to integrated and PCC requires services and roles to be re‐designed and re‐structured to be more conducive for implementation of ICDM and PCC. However, recent studies have reported that chronic care in most public hospitals in South Africa is still fragmented and patients with comorbid chronic conditions such as diabetes and HIV continue to receive care for their illnesses in separate clinics at PHC.^25^ In addition, although patient up referral for NCDs like diabetes is recommended for complications that need specialized treatment and care, health systemic issues at PHC facilities such as inadequate staff, equipment and drug stock out necessitates patient referrals to higher levels of care (E. N. Bosire, S. A. Norris, J. Goudge, E. Mendenhall, unpublished data, 2020).^26^ Due to lack of integrated services, providers struggle to manage patients with multiple chronic conditions due to poor communication amongst providers on issues around polypharmacy, lack of guidelines and decision-making tools for multiple conditions, and the difficulty of trying to manage multiple problems in a single, fixed-time consultation.^27,28^

 While the ICDM framework is being implemented in different parts in South Africa, little is known about healthcare providers experience managing patients with chronic comorbidities, and their perceptions on PCC. This study sought to investigate provider’s perspectives on PCC for patients with comorbid diabetes and HIV at a public tertiary hospital in Soweto, South Africa. We explored the challenges and opportunities that exist to practice PCC.

## Methods

###  Study Design 

This study used an exploratory research design – which aimed to gain new insights and understanding of PCC.^29^ The study was conducted between April 2018 to June 2019 and explored health and healthcare for patients with both diabetes and HIV at a public tertiary hospital in Soweto, South Africa. The first author (ENB) engaged in extensive participant observation at the clinic and conducted multiple qualitative interviews with various actors within the healthcare system. The use of both observations and in-depth interviews complimented each other and facilitated triangulation of study findings.

###  Settings and Study Population

Soweto is a peri-urban neighborhood located about 15 km south west of Johannesburg’s central business district. The township played a historically significant role in the anti-apartheid struggle and political resistance.^30^ Within Soweto, the double burden of HIV and diabetes is escalating,^31^ presenting the health system not only with a greater disease burden, but the challenge of responding to patients with comorbidities.

###  Sampling and Data Collection 

We used ethnographic methods of participant observation and in depth interviews with various stakeholders in diabetes-HIV care – from doctors, endocrinologists, dieticians, podiatrists, nurses, hospital administrators, data managers and social workers – to capture a broad and deep understanding of how care for patients with concurrent HIV and diabetes was provided. The first author (ENB) conducted observations during patient clinical encounters with providers, during patient educational sessions, or in queuing spaces. She participated in everyday activities at the clinic, watching, listening, asking informal questions, helping nurses arrange clinic rooms and sometimes help to identify patients with diabetes who were admitted in the wards. However, she did not participate in treating or managing the patients.^32^ Observations were conducted for 6 hours, 5 days in a week for the first 3 months, this was reduced to 3 days a week for the remaining months. In this article, we draw on 30 semi-structured in-depth interviews (30–60 minutes) and observations, conducted with healthcare providers. Providers were recruited using both purposive sampling and snow-ball methods. First purposive sampling was used to recruit one healthcare provider from the various professions: doctors, endocrinologists, dieticians, podiatrists, nurses, hospital administrators, data managers and social workers. These providers were recruited on the basis that they interacted with or managed patients with diabetes, HIV or both. Once interviewed, providers were asked to suggest contacts to approach for recruitment of subsequent interviews. We therefore used a snow-ball sampling method^33^ to recruit other providers, until saturation point was attained.

 Formal interviews with providers were carried out in English in a private clinic room in the hospital; questions addressed the participant’s current job, experience managing patients with diabetes and HIV, and challenges and facilitators in management of patients. The final portion of the interview addressed their perceptions around the ideas of integrated and PCC in South Africa. Providers’ responses in relation to whether they practiced PCC enabled further probing on their understanding of the concept, opportunities or barrier to practice PCC. Audio files from the interviews and field notes from observations were transcribed verbatim. All transcripts were checked against the recordings to verify accuracy.

###  Data Analysis

Data collection and analysis were conducted concurrently. Our data analysis adopted an inductive approach– drawing from a grounded theory approach. The main aim here was not to generate a new theory but to use tenets of grounded theory to develop a systematic, iterative and reflexive process through the multiple steps of the research program. We collected and analyzed data, re-evaluated and questioned insights developed, and followed leads emerging from the analysis to inform newly refined research questions, observations, and analysis.^34^ This process enabled the researcher to capture all potentially relevant aspects of integrated and PCC as soon as they were emerged. Transcripts from audio files and field notes were read repeatedly by the first author, employing an inductive approach of immersion in the text through repeated reading (and studying any drawings in a similar way), thereby developing provisional analytic categories. These categories were refined by the constant comparative method; comparisons were made across themes emerging from field notes and audio transcripts. Making comparisons facilitated challenging already grouped data with new categories and this helped the researcher to guard against bias, thus enabling consistency and precision of data. These categories were then reviewed by the second author, and any identified discrepancies were solved at this level. This involved discussions between the first and second author. Lastly, the third and fourth author were involved in discussions about the key themes that emerged from the transcripts. Once all the 4 authors were in agreement, the first author developed a codebook which was reviewed by the second author. The final codebook was uploaded in QSR Nvivo 12 software where coding was done, and emerging codes were added throughout analysis. Initially, 40 parent nodes were identified, discussed, and defined, which fell into 15 parent nodes with a number of child nodes. Subsequent reading enabled the splitting of the parent nodes to child nodes, which provided a fast snapshot of similarities, differences, patterns, and relationships from our data. The analysis led to obtaining providers,’ definition and conceptualization of PCC, their attitudes and practices of PCC and existing opportunities and barriers to practice PCC.

## Results

Thirty study participants between 25-56 years of age represented varied professional disciplines within the hospital (Table 1). Most had served for more than 10 years at the hospital. Half (n = 15) of the participants spoke both English and vernacular languages; they included nurses (n = 6), social workers (n = 2), dieticians (n = 1) and administrators and data managers (n = 4). Only 2 doctors (n = 2) spoke vernacular languages.

 In what follows, we describe 3 key themes and sub-themes that emerged iteratively from our data:

 a. Provider’s understanding, attitudes and practices of PCC

 b. Barriers to the practice of PCC in a public tertiary hospital

 c. Enabling PCC: provider’s perceived needs and recommendations

**Table 1:** Demographic Characteristics of Participants

**Characteristics**	**N = 30 (%)**
Gender	
Male	12 (40)
Female	18 (60)
Age	
25-35	9 (30)
≥35	21 (70)
Language(s)	
English only	10 (33)
English and vernacular	15 (50)
English and other(s)	5 (17)
Ethnicity	
Black	16 (53)
White	11 (37)
Indian	3 (10)
Profession	
Administrators and data managers	6 (20)
Dieticians	4 (13)
Doctor and endocrinologists	9 (30)
Nurses	6 (20)
Podiatrists	3 (10)
Social workers	2 (6)
Years of service	
≤10	11 (37)
> 10	19 (63)

###  a. Provider’s Understanding, Attitudes and Practices of Patient- Centred Care

 In this domain, we present findings on provider’s conceptualization and understanding of PCC, their attitudes and practice of PCC at a tertiary hospital in Soweto.

####  Conceptualization of Patient- Centred Care

Our findings show that providers had varied perceptions and understanding of what PCC was about. Many related PCC to the “*Batho Pele*” principles - roughly translated from the Sotho language means “people first.”^35^ The “*Batho Pele*” principles seek to introduce a new approach to service delivery that puts people first, and encapsulates the stated values of public service in South Africa, including healthcare. One nurse said:* “The patients have to come first, we have the “Batho Pele” principles which means people first. That is what we follow.” *For others, the meaning of PCC was derived from the name itself, which was understood to mean placing the patient at the centre of care and taking patient’s interests first. One doctor said, “*PCC means that the patient and his or her needs must be at the center of care.”*

PCC was also perceived to mean treating the patient as a person, a human being capable of engaging in other social events and leading a normal life in spite of illnesses:

“*We all know that they are humans, they must attend to social functions like weddings or funerals […] Sometimes it is a challenge to start requesting for special foods in such social functions. So I must understand the patient from this perspective”* (Provider 8, nurse).

Some providers, especially nurses and dieticians described PCC to encompass understanding patient’s social contexts. This included availability of resources to manage their illnesses, social support among others (see Box 1). In addition, some related PCC to empathizing with the patients:

**Observations in a Diabetes Education Session**

*“Three things are important in management of your diseases: Compliance to your medication, right diet and exercise.”* During an ongoing diabetes education session, a dietician is busy educating patients on how to manage their conditions. About 10 patients who have just finished their clinical encounter are seated at a round table. Reinforcing the dietary requirements, the dietician uses a white board marker and a pen to draw 2 columns, one with permissible foods. As she lists the different groups of foods, she engages the patients who mention different foods as she writes them on the board. The dietician uses her expertise to reinforce dietary requirements that patients should follow. Suddenly, a doctor walks in accompanied by a middle- aged male patient. He [doctor] addresses the dietician saying; *“His glucose is very high. He still uses sugar in his coffee and everything else he eats is wrong!”* [His face looks sad], the doctor walks away. The dietician continues educating patients before she picks a colored plate from the table. The plate is partitioned into required food portions. Patients’ eyes are glued on the plate as she raises it and explains what the different portions mean. She emphasizes *“You should always follow these dietary requirements: 3 meals and 2 snacks in a day.”* One male patient asks, *“But how can I afford all these foods that you are talking about? They are very expensive.”* The dietician answers; *“You may not modify your diet at once but slowly you need to change from the non-permissible foods to the permissible foods.”* The patient sits back. Immediately, another middle-aged patient asks; *“I too don’t think I can afford this […].”* The dietician says; *“I understand all your concerns. If you don’t have money for the fruits or other foods that I have talked about, you can take whatever is available at home in small portion […] take a little pap [local maize meal] In between the 3 main meals, just ensure you take something in small portions.”*

“*PCC means that you must empathize with the patient and encourage her or him to continue with the medication*” (Provider 4, nurse).

 On the contrary, other providers reported that PCC meant following treatment protocols, which focused on ethical principles of treating a patient- such as respect and empathy. Accordingly, they felt there was nothing new about PCC because they always practiced it:

“*Maybe it’s the training we receive. I usually try to treat the patient based on my professional training and experience which shows you must respect the patient” *(Provider 10, doctor).

“*I am trained to take care of patients and they [patient] also entrust their lives on us. So, I always apply these principles […]”* (Provider 1, nurse).

####  Attitudes Towards Patient- Centred Care

 Although most providers understood the importance of PCC, some especially nurses, perceived PCC to be overly representing patient’s interests and well-being without taking account of the healthcare providers as key actors in patient-provider relationships:

*“Our managers should support us too, and show appreciation. It shouldn’t just be about the patients, we are working hard, and they should also be there to support us” *(Provider 4, nurse).

*“Like I said no one wants to listen to you, it’s always the patient first. Just like now if a patient can come and says this nurse was rude to me, then you will be summoned to quality office, you are forced to say I’m sorry and nobody cares if the patient was wrong or not” *(Provider 12, nurse).

One doctor revealed how patients were taking advantage of the “ *Batho Pele”* principles when he said:

####  “We’ve got a problem of litigation and one of the loopholes is that patients are taking advantage of the “Batho Pele” policy which states people first. A patient may come in when her or his sugars are really bad and they expect you to do everything to salvage the situation, if not, they will sue the hospital.” 

####  Practices in Patient- Centred Care for Patients 

Although providers cited various ways in which they understood PCC, many endeavored to ensure that patients received appropriate education and knowledge that would facilitate self-management of their diseases. However, our findings revealed that knowledge provision was mostly done in group sessions, such as during diabetes education sessions, as opposed to a one-on-one session between provider and patients. This limited a closer understanding of the patients’ issues. The 4 key approaches that emerged as successes in providing PCC are described below: (*a*) providing collaborative care; (*b*) understanding the patient better and cultural competence; (*c*) structural competence; and (*d*) empathizing with the patients.

First, providers mentioned instances in which they provided collaborative care to patients. They mostly talked about engaging with other providers and used phrases such as *“I would try”* or “ *we are trying to”* when describing how they offered care to patients. One nurse said: *“Right now we are trying to do that [collaborative care], we include the dieticians and podiatrists so that the patients can see all the specialists.”* Such phrases show that although providers were trying to offer PCC, the process was not always easy. Some mentioned that they involved not only the patients but also their families or caregivers. In addition, providers mentioned how they viewed the patients as a whole, placing them at the centre of management as described by a one podiatrist: “ *If patients are not placed at the centre of management of their diseases, then you’ll miss out important things. For example, I can prescribe the most expensive drug, but if I only look at the diseases and not social or psychological issues, then it’s all useless.”*

Second, understanding the patients better, including their culture, languages and socio-economic status were identified as important in enhancing PCC. This was identified as a key strategy to minimize patient-provider distance. One dietician said: *“when it actually comes to the practicality of care, we have to asses if the patient can afford the required food, if not, we do refer them to the social workers to take action.”* On occasion, providers would deviate from the guidelines and accommodate patients’ treatments or to take stock of their social realities (see Box 1). Many described how being culturally competent enabled them to work well with the patients because, the patients felt much respected. One nurse said; *“Some of these patients strongly believes in cure from traditional healers and spiritual people, it depends on what their culture is all about. So I can’t ask them to stop their beliefs because, if I do so, they will default their appointments.”*

Although many variations exists in literature in definitions of cultural competence,^36^ it is widely acknowledged that cultural competence is the attitudes, knowledge, and skills necessary for providing respectful and quality care to diverse populations.^36,37^ However, researchers have paused to ask; is cultural competency the same as cultural sensitivity or awareness? For instance, when a nurse achieves cultural competency with a specific culture, is the nurse now culturally competent with all persons of that particular cultural background^38^? This notion, that through education, providers can truly understand the lived experience of patients, has proven problematic. Others have argued that cultural competence may be perpetuating stereotypes about what members of a particular “culture” believe, do, or want, and how they should be dealt with.^39^ As such, a shift towards concepts such as cultural humility and structural competence is recommended.^37^

Cultural humility is “the ability to maintain an interpersonal stance that is other-oriented (or open to the other) in relation to aspects of cultural identity that are most important to the [person]^40^” Cultural humility focuses on self-humility rather than achieving a state of knowledge or awareness.

Structural competence emphasizes recognition of the economic and political conditions that produce health inequalities in the first place.^41^ The concept pushes the needle on what competence means – unlike cultural competence, structural competence begs the provider to think in terms of structural challenges – such as income, location, access, etc as opposed to beliefs or culture.

 Thus, providers in this study described how recognizing structural barriers (economic and political conditions that produce inequalities in health) to patients’ abilities to follow clinical recommendations was a fundamental way for providers to demonstrate they understood their patients’ needs.

Sometimes, nurses in the diabetes clinic used their own money to buy some loaves of bread and milk during clinic days to feed the patients who had nothing to carry from home. Although neither an adequate nor a sustainable solution to deal with patients’ structural vulnerabilities, such charitable acts were said to make the patients feel appreciated and also, reduced the divide between providers and patients, something that fostered PCC. Elsewhere, this has been referred to as “doing with the patient”^42^; where providers work closely with the patients, while understanding their needs and empower patients to make informed and practical choices. This is opposed to “doing to” or “doing for” the patients, where providers follow strict clinical guidelines when managing patients with little understanding of patients’ social needs.

Third, many providers described empathizing with patients through listening to them and giving them time to talk, especially when their health seemed to be deteriorating. This was said to facilitate a good rapport between providers and patients. In addition, most providers, especially nurses, were culturally sensitive to the patients they attended to. For instance, during one observation in the clinic; a woman in her late 50’s said; “ *I don’t believe that diabetes can be managed in the hospital, only God can heal me.”* A nurse quickly concurred with the patient and she commended the patient for trusting God, a factor that was seen to encourage the patient. A summary of examples of practicing PCC is shown in Table 2.

 Despite their efforts in practicing PCC, providers mentioned many challenges that inhibited the quality of care they provided to the patients and practice of PCC as discussed below.

**Table 2:** Examples of Patient-Centred Practice

**Themes**	**Excerpts**
Collaborative care and putting patients at the centre of care	*“I’ll discuss with the patients, I will counsel them to make sure they understand, and then I'll reinforce or get the nurses to do it” *(Provider 3, doctor). *“We would try and find out if the family members are available for us to discuss simple things like, are they able to take care of the patient […]”* (Provider 21, podiatrist).
Understanding the patient better and respect for patient autonomy	*“So, you need to know if the patient is going to dress their wound at home, is there anyone who can assist in taking care of the patient […]” *(Provider 14, podiatrist). *“You know, you have to respect the patient's rights and their beliefs and their cultures. If they communicate that they want to seek alternative care, we try and inform them of the risks of doing so but respect their wishes should they want to. And we will never deny them treatment should they come back” *(Provider 22, endocrinologist).
Structural competence	*“Patients are not able to attain good health because of lack of money to buy the required food, I do teach them what is required as per the nutritional guideline but sometimes end up giving them alternatives that are practical in their settings” *(Provider 28, dietician).
Empathizing with the patients	*“Like she is smart, she will tell me the truth that ‘I did not inject because it’s sore […]’ and I would feel for her” *(Provider 1, nurse). *“I always feel for them because I know it’s not easy. I would change their appointment dates to what fits them” *(Provider 4, nurse).

###  b. Barriers to the Practice of Patient- Centred Care in a Public Tertiary Hospital 

Many providers described feeling powerless to provide adequate care for patients with comorbid diabetes and HIV. In many cases, provider narratives involved personal frustrations they experienced by failing to deliver higher quality care because of systemic failures. For instance, a doctor in her mid-forties exclaimed, * “I feel inadequate when I see patients dying, I am well trained to manage these patients, but I don’t have the right tools to do my work.”* As such, providers used words such as being *“frustrated,” “incapacitated”* or * “inadequate”* when describing how they were rendered powerless in care provision:

####  i. System Failures

These frustrations were exemplified in the lack of equipment or frequent malfunction or breakdown of diagnostic services. Providers were often frustrated because they had no control over equipment failure or lack of availability of treatments for their patients. For instance, an endocrinologist said; *“Sometimes you can diagnose a condition, but the treatment is not available. So what good have you actually done to the patient?”* Another doctor expressed; *“I got really frustrated in the service delivery for not being able to do everything in my power to best serve my patients because of limited resources, we are running out of drugs.” *For some, it was lack of equipment for specific tests that made it impossible to offer clinical advice for common conditions; * “We don’t have the cholesterol machine, which is important. We can’t offer advice on what they should avoid eating, we can’t help.” *In these cases, lack of specific equipment had enormous impacts on the care people received.

Many physicians expressed concern about the use of clinical guidelines for people with comorbidities or multi-morbidities, which were generally developed for single clinical conditions. Use of multiple guidelines to manage patients was said not only to be frustrating to providers but also to the patients. One endocrinologist explained; *“The guideline may indicate how to manage a patient with diabetes and hypertension or other close related comorbidities but does not take account of those with multi-morbidities or complex diseases.”* Another doctor said; *“Sometimes, I am not sure what advice to give to patients due to lack of guidelines for comorbidities.”* Lack of comorbidity guidelines was exacerbated by poor communication between providers; where most providers managed patients with comorbidities based on their own discretion, with little cross-talk amongst them. This situation was evident especially when patients were sent to meet with various providers for different health conditions.

Our findings also show that care for patients with comorbid diabetes and HIV was fragmented with lack of integration in terms of administrative and consultative functions. Patients attended different clinics as exemplified by a nurse; “ *We see the patients in different clinics. They will visit this clinic for diabetes and go to the other clinic for HIV/AIDs.” *This kind of fragmented care, characterized by physical separation of space, was partly due to the design and structure of the tertiary hospital, which focused more on providing specialty care. This limited provision of PCC. One doctor explained; *“It is even worse when they have other complications which may force them to see a dietician, podiatrist, or an ophthalmologist.”*

Observations in the diabetes clinic revealed the strain caused by staff shortages and heavy workloads. It was evident that most clinics were overstretched, exemplified by long queues and waiting time for the patients. The long queues were attributed to both patients coming earlier than expected and staff shortages. One nurse reported that; *“the patients come early and start queuing outside the clinic.”* As a result, the long queues added more pressure to providers who were forced to rush the patients through, just to clear the queues. Second, rampant staff shortages in most clinics exacerbated the issue of long queues; a doctor explained that: * “we have to treat patients in the ward first and by the time we come back to the outpatient clinics, the queues have built up*.” These sentiments were also echoed by one hospital administrator; “ *The greatest challenge that we have is staff shortages, this explains why the queues are long in most clinics.”*

Many providers described that staff shortages had a large impact on the limited time available for them to engage in lengthy discussion with the patients. This hindered them from understanding their patients fully: “ *I know I must take at least 20 minutes but due to long queues, I take about 5-7 minutes; which is not sufficient in getting to understand the patient.” *Moreover, many providers stated that staff shortages were linked to clinicians quitting their jobs. One doctor mentioned, “ *They [doctors] are always on a transit,” *leaving for better opportunities. Another middle-aged dietician described this: *“The challenge is today patients see this doctor, tomorrow another doctor […]. Doctors resign or look for better opportunities.” *Equally, during an in interview with a doctor, she fought her tears back when she said, * “I am tired of working in such conditions; overworking, no equipment, patients dying […]. The best thing is to quit.”*

####  ii. Patient Barriers

 Many providers also described how the patient-provider divide impeded them from providing PCC. With vast differences between patients and providers in income, education, culture, and ethnicity, many providers felt they could not fully understand or deliver care or advice that would be attainable by their patients. The sub-themes that emerged in this domain includes: culture, class, language and patient’s socio-economic realities as discussed below:

####  Culture, Ethnicity and Language

Cultural factors are crucial to diagnosis, treatment, and care. They shape health-related beliefs, behaviors, and individuals’ value.^43^ This study drew from both patients’ and healthcare provider’s cultures – arguing that not only patients and their communities have cultures, but that there is also a “culture” of medicine,^43^ and both cultures can hinder or foster healthcare seeking and provision. Patient’s cultural beliefs and practices impacted on the practice of PCC. For instance, a middle-aged podiatrist narrated his concern with the impact of patient preferences or self-care once they left the hospital: *“I used the most expensive dressing for the patient’s wound but when he went home, he visited the sangoma [traditional healer] who asked him to remove the dressing to allow the wound to dry. This can be so disappointing but what can I do?” *In this case, providers were faced with tension between respectful support for autonomous agency and the promotion of particular clinical goals based on their training (biomedical culture). Furthermore, many described patients’ educational status as influencing the seriousness and attitudes they expressed in managing their diseases. For instance, one nurse stated, “ *some are not educated, others don’t see the need to come back for appointments or take our instructions seriously.” *As such, some acknowledged that it was difficult to strike a balance in their efforts to enable or activate the patients. Sometimes, [provider said that] patients concealed or were reluctant to provide potentially useful information as said by one nurse; *“They [patients] lie a lot. They will intentionally provide wrong glucometer readings in their diary, you will be surprised.”* This hampered providers from getting a clear understanding of patient’s thoughts or feelings.

Providers also revealed that some patients were demotivated to engage in treatment goals or plans as revealed by a nurse; “ *we encourage them to join the diabetes school but some feel it’s a waste of time.” *Despite the few who were demotivated, some patients were more motivated and worked hard towards achieving pre-set goals. One endocrinologist said; “ *Some patients themselves feel motivated and they want to join management program. One patient moved from 135 kilos to like 80 kilos.”*

South Africa is an ethnically diverse nation, which provides a complex and intriguing picture of multilingualism. The country has 11 official languages, namely: Sepedi, Sesotho, Setswana, siSwati, Tshivenda, Xitsonga, isiNdebele, isiXhosa and isiZulu, English, and Afrikaans.^44^ In Soweto, most people speak 1 or 2 of the 9 Bantu languages listed first, and few use English and Afrikaans. Given the divergence in backgrounds between patients and providers, language barriers were constant and challenging during clinical encounters, similar findings have been reported elsewhere.^45^ Moreover, many patients visiting the tertiary hospital outside of Soweto were referred from both within and outside South Africa. Most patients speak limited English, preferring African languages that few providers speak. Most doctors, endocrinologists and dieticians spoke exclusively in English, with few who could speak African languages. Nurses and social workers spoke African languages but this did not cover the diversity of languages patients spoke. One nurse explained, *“we offer good education in our clinics, but we are not really checking whether they understand it or not.” *In addition, a dietician said, *“Especially patients from Mozambique, they are the ones that really, really are finding it difficult to understand what we are saying.”*

####  Patient’s Socio-Economic Realities

Patient’s socio-economic status – including employment, availability of food, transport to the hospital, or living environment, influenced how they adhered to their treatment regimes and also, the care they received from providers. Furthermore, these factors were said to largely hinder patient-provider partnership in care and hence, diminished practice and success of PCC. For example, providers felt powerless when they experienced patients who could not adhere to clinical advices due to unaffordability of the recommended diet. One dietician said, “ *It is so frustrating for me when I design the right nutritional plan for the patient, then the patient cannot afford.” *Patients often failed to show up for clinical appointments. Nurses would often shout at patients who defaulted their appointments. Yet, many described this was due to lack of transport to the hospital or, as one doctor explained, *“The old people will be telling me that I don’t have money, there is no one to bring me.” *Another endocrinologist said, *“Sometimes they would say that they were sick, or they had to travel for a family funeral […].” *Similarly, a podiatrist stated that: *“some of them come from far places, like Lesotho or Limpopo, defaulting is because of the distances they travel to see us.” *Defaulting clinical appointments hindered continuation of care and follow-up, on patients’ progress in managing his/her illnesses. It is with these barriers that many providers (especially those who were keen on practicing PCC) became the most frustrated. Yet, the limited flexibility in patient scheduling and appointments was difficult. This was even more complicated for patients with multi-morbidities who had to schedule and attend more than 2 clinical visits. This contributed to some patients missing appointments and showing up when least expected as described by a nurse:

####  “The challenge is, we don’t have a flexible appointment dates due to staff shortages. We see type twos [T2DM] on Monday and Thursday, and type ones [T1D] on Tuesdays […]. Imagine I have 200 patients coming in today and 20 defaulters shows up. That means we will see 220 patients that day. This creates workload for us.”

 Thus, both institutional limitations and patient’s social economic factors created a social distance between providers and patients, a factor that many described as impeding them from practicing PCC.

###  c. Enabling Patient- Centred Care: Provider’s Perceived Needs and Recommendations

Despite the existing opportunities and limitations to practice PCC, providers cited different strategies that can be employed to enable practice of PCC. First, it was mentioned that a proper implementation of integrated care could enhance practice of PCC. One nurse said: *“I think the best way to approach PCC will be to manage all the condition in totality, we shouldn’t just concentrate on one. We must bring everything in, there should be social workers coming, doctors and all other providers.” *Another doctor recommended strengthening of PHC facilities when he said *: “But this hospital [tertiary level] is not the right place for doing integrated care. Primary centres need to be strengthened to provide integrated care.”*

In addition, providers also cited that addressing the issue of staff shortages and providing necessary equipment would enable them to practice PCC efficiently. One doctor said; * “These challenges wouldn’t be as bad if we had enough monitoring equipment. That is a huge challenge I experience everyday and it need to be addressed”* her sentiments were echoed by one endocrinologist who said: *“So infrastructure is a big problem, we don’t have enough resources for us to manage patients well. […] the government should look into this.”*

Providers also mentioned the need for more training on how to offer integrated and PCC. One doctor recommended that providers working at PHC clinics must be trained using a family physician approach: *“As a South African doctor, for the majority of the time you are trained by a family physician and this is the model they should be applying because it is more integrated. So in the primary health sector, I would expect providers to be trained using similar approach.”* Another doctor concurred when he said: *“Yes, definitely the knowledge needs to be improved quite a bit. Also, we do not have a good training program, the nurses need more training on PCC given the number of patients they see […].” *Providers mentioned that there was a need to train patients on the importance of PCC, while stressing out the importance of patients understanding their roles and responsibilities in chronic care. Others mentioned that it was important to conduct community awareness on NCDs so as to prevent these diseases at the first place: *“More information has been provided on HIV and TB, but most patients don’t know about diabetes and its management. At the moment I would say knowledge is very limited at the community level and people must be educated and trained on how to prevent these diseases.”*

## Discussion

This is the first study to employ ethnographic methods to investigate healthcare providers’ perspectives and practices of PCC for patients with comorbid diabetes and HIV in Soweto. First, providers mentioned different ways in which they conceptualized PCC, most relating it with the *“Batho Pele”* (people-first) principles. Others understood PCC to mean placing the patient at the centre of care, empathizing with the patients or treating patients as a person. Second, this study found that the practice of PCC was hampered by both health systemic barriers and patient-related factors. Health systemic issues such as lack of equipment and guidelines for multi-morbidities, staff shortages and the structure of the tertiary hospital which promoted specialty care as opposed to integrated care, diminished possibility of practicing PCC. Patient-related factors such as language barrier and poverty constrained their involvement in decision-making pertaining to their disease management. In most cases, the practice of PCC was inconsistent as patients were activated in group sessions with no further personalized engagement with providers that would facilitate interactions and partnership in care. Finally, our findings show provider perceived needs and recommendations that might enhance their practice of PCC in Soweto. We will discuss these points in turn.

###  Provider Understanding, Attitudes, and Practices of Patient- Centred Care

Providers largely related PCC to the *“Batho Pele”* principle, one of the South African government public service policies, which means “people first” and aims on transforming public service delivery.^35^ Our findings are in line with earlier studies in South Africa, which have reported that nurses equated PCC to practicing *“Batho Pele”* principles.^46^ In this context, providers narrated how they respected the patients and considered their views in care. Others derived their understanding of PCC to mean placing patient at the centre of care. Unlike the “ *Batho Pele*” principle that considered patients first (by respecting and prioritizing their needs), placing patients at the centre of care was said to be above and beyond this: It ensure that patients were treated as a whole – here, providers not only looked at patients’ illnesses but also, tried to understand their socio-economic issues that were outside the purview of medicine. In addition, some providers mentioned that PCC meant empathizing with the patients and treating them as persons or humans capable of making informed decisions. Our findings parallel earlier studies which have pointed out that the concept of PCC can be variously interpreted.^20,21^ However, there is general agreement that PCC broadens the conventional medical approach to include the patient as an active participant in his or her care, and to promote the physician-patient partnership.^21^ Although most conceptualizations were aligned with PCC as reported in literature, few providers deviated from the key principles of PCC; their understanding of PCC was about implementing knowledge from their medical training and following clinical guidelines and protocols. Such an understanding is not fully congruent with the PCC constructs described in the literature. For the others, they perceived PCC to be overly representing patient’s interests and well-being without taking account of the healthcare providers’ needs. Thus, there is need for provider training on the key tenets of PCC.

###  Barriers to practice of Patient- Centred Care in Soweto

First, providers experienced frustrations, inabilities, and burnout when managing patients due to the poor working conditions characterized by staff shortages, workload, and insufficient equipment; factors that constrained their abilities to deliver high-quality care to patients. Our findings validate earlier studies which have reported how hospital shortages and poor working conditions have limited providers from managing patients with comorbidities in South Africa^26,47^ and in other low- and middle-income countries.^48^ In addition, such factors have been frequently mentioned as major barriers to implementation of PCC.^40,50^ Due to inadequate resources and overstretched tertiary level hospitals in South Africa, decentralization and integrating diabetes care into PHC facilities may reduce such challenges. Although the ICDM has been implemented in most PHC clinics in South Africa, the implementation is not smooth across all provinces; places like Soweto continue to experience slow implementation rates due to structural challenges such as lack of medication and staff shortages. There is need for the South African Department of Health to ensure that these challenges are addressed for successful integration of diabetes care at PHCs. Decentralization of chronic care will facilitate closer working relationships between patients, nurses and community healthcare workers at PHC, thus improving patient clinical attendance, clinic functioning, understanding the patient better and easier implementation of PCC.^51^

Second, lack of guidelines for comorbidities and multi-morbidities, with none extending to address patient’s cultural or social realities compounded providers’ frustrations. Earlier studies have reported that most guidelines are generally written for managing sole conditions and do not account for the unique circumstances of each patient.^52,53^ Furthermore, this study revealed that patients attended separate, fragmented clinics, a finding that has been reported in earlier studies.^25,54^ Inadequacies of using a single guideline for comorbidities and patients visiting separate clinics limited provider shared decision-making and flexibility to respond to patient’s complex, personal experiences. Instead, this study shows that guidelines should accommodate conditions that cluster together, while supporting providers to deliver a more personalized, integrated and comprehensive PCC.

Third, this study conveys a complicated story around how patient social challenges such as financial insecurity, unaffordability of healthy diets, and lack of transport to the hospital impeded providers’ ability to offer quality care to patients and further hampered practice of PCC. Previous studies have shown how unaffordability of clinically recommended foods especially for people with diabetes^55^and patients’ lack of transport to the hospital^56^ has led to poor health outcomes. Indeed, this finding underscores how significant structural competence can be for clinical care in contexts like Soweto. As opposed to cultural competency, which can reify cultural stereotypes, Metzl and Hansen have argued that structural competence emphasizes how structures shape clinical interactions, develop deeper structural divides within and between patient-provider relationships, and beg for interventions and understanding of structural divides as well as structural solutions for care.^41^ Harris and colleagues have pointed out that patients are happier, more cooperative and productive, and more likely to make positive changes in their behaviour when providers do things with them, rather than to them or for them, which is a key tenet in PCC.^42^ As revealed in our study, although health systemic challenges and patient social challenges hindered the practice of PCC, some providers (especially dieticians) deviated from clinical guidelines for managing patients to discuss with the patients on the practicality of care they designed (see Box 1). This was based on patients’ social and economic circumstances; thus designing care involved engaging the patients rather than designing care for them. We recommend that clinicians should consider the upstream determinants of health if PCC is to be practiced in resource-constrained settings like Soweto.

Unsurprisingly, language barriers severely limited physician-patient communication and engagement in care. Our findings concur with recent studies which have elucidated how the language barrier remains a crucial challenge to providing equitable and quality healthcare to patients in South Africa^57,58^; thus limiting patient participation in care.^59^ Although it is easy to explain these systemic problems away by highlighting the linguistic diversity of Soweto, there are multiple employable interpreters, nurses, clerks, and other staff who either already speak many of the languages that doctors do not. In some ways, this is an upstream problem that highlights the need to identify and incentivize medical institutions to encourage multi-lingual South Africans to train in the field and fill these roles after completing medical school, as evidenced in other settings.^60^

###  Provider Perceived Needs and Recommendations

Providers recommended strategies that would enhance successful implementation of PCC. First, many reported that PCC was more practical at PHC clinics rather than at tertiary hospitals. Understanding this disjuncture is critical – why might it be difficult to focus on patient needs within the tertiary hospital setting? Perhaps, within hospitals, shifting from focusing primarily on the specialist, to the needs of the patients, would provide an opportunity for more integrated HIV and diabetes care – even in the most serious of cases. Many also cited that there was a need to address staff shortages and provide necessary equipment at hospital. Furthermore, some recommended further training for providers on how to better provide PCC. In practice, providers sometimes find it hard to shift from biomedical approaches of care to more person-centred approaches.^61^ This calls for training and equipping healthcare professionals (especially in medical schools) with both cultural humility^37^ and structural competence skills that can promote the change that is required to accomplish PCC in South Africa.

Providers reported a need to provide training or education to patients – so as to understand their role as partners in care and be willing to collaborate with providers as well as share their self-care experiences and concerns.^22,23^ However, it is important to note that within the bureaucratic system of a large hospital, it’s difficult to place the burden of systemic transformation on patient advocacy alone. These unexhaustive recommendations taken together may strengthen the practice of PCC in public health facilities in South Africa.

###  Study Limitations

 This study is not without limitations. First, this study did not involve patients, thus lacking the patients’ voice. Second, all participants were drawn from one tertiary hospital in Soweto, and experiences at PHCs even in Soweto may differ. Therefore, it’s unclear whether all patients sent to receive care at the tertiary hospital were experiencing complications, or if it was serving as an alternate PHC option. Third, findings from this study may not reflect clinics in other provinces in South Africa. Nevertheless, the findings provide important insights for those managing patients with comorbid diabetes and HIV because even in one of the best public hospitals, care is not integrated across conditions that people encounter. In addition, participant observation and in depth interviews with healthcare providers across the health system provided an in depth study of integrated care possibilities and pitfalls within this very specific context.

## Conclusion

 Policies advocating for integrated care and PCC for people with comorbidities and multi-morbidities in South Africa may fail to achieve the intended outcomes if health systems do not work toward alleviating institutional limitations and addressing the challenges experienced by healthcare providers. There is a need for healthcare systems to change approaches that meet the challenges and complexity of patients with comorbidities or multimorbidity by developing guidelines that take account of these conditions. It is important to strengthen PHC clinics in South Africa by ensuring access to necessary resources that will facilitate successful integration and management of comorbid diabetes and HIV. Training medical practitioners and students on PCC, and equipping them with cultural humility and cultural competence skills may also enable a better understanding of patients in their socio-cultural contexts. Lastly, training patients is also key in ensuring they too, understand their roles as partners in chronic care, and they should be willing to collaborate with healthcare providers in care and treatment.

## Acknowledgements

 We wish to thank all our participants for their sincere responses on their experiences in managing and caring for patients with comorbid diabetes and HIV/AIDS in Soweto. We also thank the staff and management of the diabetes/endocrine clinic at the tertiary hospital in Soweto for their support during this study. Finally, we thank Sonja Klingberg at the Developmental Pathways for Health Research Unit, University of the Witwatersrand for proofreading and thoughtful comments on this manuscript.

## Ethical issues

 Written informed consent was obtained from the study participants after reading out the content of the information sheet and explaining the purpose of the study. This study was approved by the tertiary hospital’s research committee and the Human Research and Ethics Committee at the University of the Witwatersrand, Johannesburg, South Africa (Ethics number: M171125).

## Competing interests

 Authors declare that they have no competing interests.

## Authors’ contributions

 ENB conceptualized, conducted the study, analyzed data, and wrote the manuscript; EM, SAN, and JG conceptualized the study, provided supervision, and reviewed and approved the manuscript.

## Funding

 The South African Medical Research Council funded this study from the core grant to DPHRU (SHNS017-NORRIS S MRC 2017-NORRIS S MRC 2017). ENB is supported by the South African Medical Research Council. SAN is supported by the DST-NRF Centre of Excellence in Human Development at the University of the Witwatersrand, Johannesburg, South Africa.

## Authors’ affiliations

^1^South African Medical Research Council Developmental Pathways for Health Research Unit (DPHRU), School of Clinical Medicine, Faculty of Health Sciences, University of the Witwatersrand, Johannesburg, South Africa. ^2^Science, Technology, and International Affairs Program, Walsh School of Foreign Service, Georgetown University, Washington, DC, USA. ^3^School of Human Development and Health, University of Southampton, Southampton, UK. ^4^Centre for Health Policy, School of Public Health, Faculty of Health Sciences, University of the Witwatersrand, Johannesburg, South Africa.

## Key Messages

**Implications for policy makers**

- This study emphasizes the need to strengthen health systems in South Africa and other similar contexts, by increasing the number of staff and developing multidisciplinary guidelines for managing comorbidities. This would also facilitate implementation of integrated and patient-centred care (PCC).
- Policy-makers can improve care for people with comorbid diabetes and HIV by ensuring sufficient equipment and trained staff at primary healthcare (PHC) clinics in South Africa.
- Medical institutions need to invest in training and equipping medical practitioners and students with cultural humility and structural competence skills that would help them adopt PCC.

**Implications for public**

While this research focuses on healthcare providers’ perspectives, and not directly on the patients, the findings demonstrate a need for a close relationship between healthcare providers and patients in terms of chronic disease management. Patients’ socio-cultural and economic backgrounds cannot be ignored within clinical spaces. As such, patients’ voices, perspectives and lived experiences of chronic diseases are important especially when designing chronic care programs that are contextually appropriate. Providers need to support patients with and often also, their informal carers’ in improving their knowledge and skills and encouraging them to actively participate and collaborate with providers in decision-making and self-management.
